# Putting Warburg to work: how imaging of tumour acidosis could help predict metastatic potential in breast cancer

**DOI:** 10.1038/s41416-020-01171-2

**Published:** 2020-12-01

**Authors:** Michala Gylling Rolver, Stine Falsig Pedersen

**Affiliations:** Section for Cell Biology and Physiology, Department of Biology, Faculty of Science, University of Copenhagen, Copenhagen, Denmark

## Abstract

Solid tumours are often highly acidic compared to normal tissue, and tumour extracellular acidosis contributes to multiple aspects of cancer progression. Now, Anemone et al. in this issue of the *British Journal of Cancer* provide in vivo evidence that the degree to which various breast cancer cell lines acidify their environment correlates with their ability to metastasise to the lungs. This indicates that measurements of tumour extracellular acidosis have the potential to become a clinical tool for assessing the risk of metastasis.

## Main

It has been known for decades that the tumour microenvironment is acidic.^1,2^ This can be partially ascribed to the Warburg effect—the characteristic glycolytic shift of many cancer cells even in the presence of oxygen, resulting in copious H^+^ and lactate production. More precisely, however, this excessive extracellular acid stems from the combined contributions of both H^+^ from glycolysis and acid in the form of CO_2_ from oxidative metabolism, which are extruded from the cancer cells and accumulate in the torturous, insufficiently vascularised tumour interstitial space.^3^ Despite their elevated metabolic acid production, the intracellular pH (pH_i_) of cancer cells growing at an acidic extracellular pH (pH_e_) is less acidic than that of normal cells under similar conditions, because the cancer cells greatly increase their capacity for acid extrusion.^4^ Importantly, this not only helps them survive the potentially toxic acidosis, but can endow cancer cells with additional advantages, including chemotherapy resistance, immune evasion, and increased invasiveness and metastasis.^4–6^ Collectively, this strongly suggests that the ability of cancer cells to acidify their environment is related to their aggressiveness, rendering precise tumour pH_e_ measurements highly clinically relevant. However, most methods for in vivo pH imaging are either of relatively low resolution, lack the ability to distinguish pH_e_ and pH_i_ or are not safe for clinical use. These include microelectrodes, which are invasive and imprecise, positron emission tomography approaches, which report on a composite of pH_e_ and pH_i_, and genetically encoded or injectable optical imaging techniques, which are often not clinically relevant and remain limited by the lack of sufficiently specific near-infrared pH_e_ probes.^1,2^

In this issue of the *British Journal of Cancer*, Anemone et al.^7^ used magnetic resonance imaging (MRI)-based, pH-sensitive chemical exchange saturation transfer (MRI-CEST)^8,9^ to demonstrate that extracellular acidosis correlates with tumour metastatic behaviour in vivo. Employing spontaneous BALB-neuT mammary tumours as well as syngeneic engrafting of metastatic and non-metastatic mammary cancer cell lines in BALB/c mice, they created spatial pH_e_ maps of primary tumours and defined an acidity score highlighting pH_e_ heterogeneity. They found tumour pH_e_ to be profoundly acidic, below 6.8 in the most aggressive models, and less acidic but still below pH 7, in the less aggressive tumour models. Supporting earlier reports,^10,11^ they demonstrated substantial heterogeneity of tumour pH_e_. The acidity score, albeit not the average tumour pH_e_, correlated significantly with the number of lung metastases. This appeared to at least partly reflect cancer cell-autonomous properties, as invasiveness also correlated inversely with pH_e_ in vitro.

While earlier studies have proposed a correlation between invasiveness and extracellular acidity in tumours,^12,13^ the work by Anemone et al.^7^ takes this an important step further by combining the precision and clinical potential — MRI-CEST has been used in patients^14 ^— of MRI-CEST with studies of mammary cancer phenotype. Notably, they found that the tumour acidity score correlated not only with metastasis, but also with the expression of cancer stem cell markers in the tumour tissue, extending earlier reports that stemness in glioma models is dependent on an acidic niche pH_e_.^15^ To further interrogate the relation between pH_e_ and metastatic capacity, Anemone et al. used a complementary approach: they adapted 4T1 cells to growth at chronic acidosis and showed that, although the primary tumours formed by the acid-adapted cells grew more slowly than their normal pH counterparts, they acidified the tumour microenvironment more, and, concomitantly, gave rise to an increased number of lung metastases. Interestingly, in this case, this was not recapitulated in vitro, where the acid-adapted cells were less invasive than wild-type cells. This demonstrates that for reasons that are still elusive, the correlation between acidosis and invasive/metastatic behaviour is context-dependent and apparently stronger in vivo than in vitro.

The most pertinent open question in the study is that of causality. In other words, to what extent does the correlation between extracellular acidity and metastatic potential reflect a driver role for extracellular acidosis per se, and to what extent does it simply happen because cancer cells with very high metabolic acid production and a corresponding need for net acid extrusion are also the most aggressive ones? Acidosis is clearly favourable for extracellular matrix degradation, and observations such as the metastatic behaviour of acid-adapted cells observed in this^7^ and other^5,6^ studies are clearly indicative of some degree of causality. However, it must be stressed that pH_e_ impacts pH_i_, and that acidic pH_i_ is growth inhibitory, even for cancer cells.^4^ Thus, it seems likely that additional factors, such as effects of pH on anti-cancer immune response and vascularisation,^4^ may contribute in the in vivo setting, and we are still far from a full understanding of the complex pro- and anti-tumorigenic effects of acidic tumour pH_e_. It would therefore probably be naive to think that simply making tumours less acidic overall will be a magic bullet for reducing metastatic potential. Indeed, studies in which tumour pH_e_ was increased by oral HCO_3_^−^ administration have shown opposing effects on metastasis in different models.^16,17^ Curiously, HCO_3_^−^ treatment failed to significantly alter tumour pH_e_ in the model used by Anemone et al., and if anything, slightly increased the number of lung metastases. Another approach would be to directly target net acid extruding transporters. Supporting the therapeutic potential of this, such transporters are frequently upregulated in cancer cells, and their inhibition or knockdown has been shown to reduce primary tumour growth^18^ as well as in vitro invasion.^19^

Another key open question is the relation between acidic pH_e_ and other hallmarks of the tumour microenvironment in regulating metastatic behaviour. For instance, microenvironmental hypoxia and acidosis can exist both independently and in an overlapping manner in tumours,^10,11^ yet the impact of varying combinations of these factors is essentially unstudied. MRI-CEST can already be combined with other clinical imaging modalities,^8^ and, for instance, combined pH- and hypoxia mapping in tumours could be a highly informative tool, both in basic research and translated to a clinical setting. As such, the study by Anemone et al. is an early step, but an important one. While the sample studied is yet too small to provide evidence of a global correlation, let alone causality, between pH_e_ and metastasis, it clearly shows that clinically relevant mapping of tumour pH_e_ with high spatial resolution has the potential to predict cancer aggressiveness. This opens for the possibility that tumour pH_e_ imaging could once become a valuable tool for predicting metastatic potential in a clinical setting.

## Data Availability

Not applicable.
